# Ethyl 2-isopropyl­amino-6-methyl-8-oxo-3-phenyl-3*H*,8*H*-furo[2,3-*d*][1,2,4]triazolo[1,5-*a*]pyrimidine-7-carboxyl­ate

**DOI:** 10.1107/S1600536810032654

**Published:** 2010-08-25

**Authors:** Qing Li, Xian-Yu Wang, Cheng-Ming Qin, Yan-Lin Wang

**Affiliations:** a Department of Anesthesiology, Zhongnan Hospital of Wuhan University, Wuhan 430071, People’s Republic of China; b Department of Anesthesiology, Taihe Hospital of Hubei Medical University, Shiyan 442000, People’s Republic of China

## Abstract

In the title compound, C_20_H_21_N_5_O_4_, the ring system containing the three fused rings is essentially planar (r.m.s. deviation for all 12 non-H atoms = 0.041 Å). The phenyl ring makes a dihedral angle of 54.41 (6)° with this ring system. The isopropyl group is disordered over two positions, with site-occupancy factors of 0.753 (9) and 0.247 (9). The structure is mainly stabilized by weak inter­molecular N—H⋯O and intra­molecular C—H⋯O hydrogen-bonding inter­actions and π–π inter­actions, with inter­planar distances of 3.415 (1) Å between adjacent furan ring centroids and 3.420 (1) Å between the benzene and pyrimidinone rings.

## Related literature

For the crystal structures of other fused pyrimidinone derivatives and related literature, see: Ding *et al.* (2004 ▶); Hu *et al.* (2005 ▶, 2006 ▶, 2007 ▶, 2008 ▶).
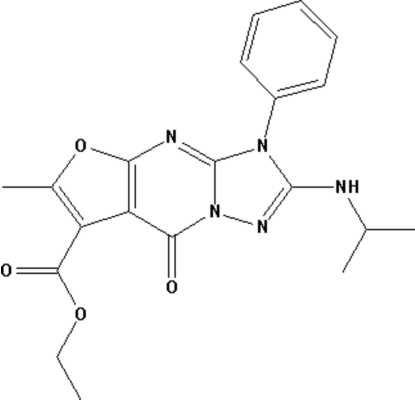


## Experimental

### 

#### Crystal data

C_20_H_21_N_5_O_4_
*M* *_r_* = 395.42
Orthorhombic, 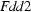

*a* = 19.9810 (11) Å
*b* = 37.3673 (19) Å
*c* = 10.7181 (6) Å
*V* = 8002.5 (7) Å^3^
*Z* = 16
Mo *K*α radiation
μ = 0.09 mm^−1^
*T* = 295 K
0.20 × 0.20 × 0.10 mm

#### Data collection

Bruker SMART 4K CCD area-detector diffractometer
Absorption correction: multi-scan (*SADABS*; Sheldrick, 2003 ▶) *T*_min_ = 0.981, *T*_max_ = 0.991
21970 measured reflections
2074 independent reflections
1883 reflections with *I* > 2σ(*I*)
*R*_int_ = 0.042

#### Refinement

*R*[*F* ^2^ > 2σ(*F* ^2^)] = 0.043
*wR*(*F* ^2^) = 0.109
*S* = 1.09
2074 reflections
290 parameters
5 restraints
H atoms treated by a mixture of independent and constrained refinement
Δρ_max_ = 0.14 e Å^−3^
Δρ_min_ = −0.11 e Å^−3^

### 

Data collection: *SMART* (Bruker, 2001 ▶); cell refinement: *SAINT-Plus* (Bruker, 2001 ▶); data reduction: *SAINT-Plus*; program(s) used to solve structure: *SHELXS97* (Sheldrick, 2008 ▶); program(s) used to refine structure: *SHELXL97* (Sheldrick, 2008 ▶); molecular graphics: *PLATON* (Spek, 2009 ▶); software used to prepare material for publication: *SHELXTL* (Sheldrick, 2008 ▶).

## Supplementary Material

Crystal structure: contains datablocks I, global. DOI: 10.1107/S1600536810032654/bt5323sup1.cif

Structure factors: contains datablocks I. DOI: 10.1107/S1600536810032654/bt5323Isup2.hkl

Additional supplementary materials:  crystallographic information; 3D view; checkCIF report

## Figures and Tables

**Table 1:** Hydrogen-bond geometry (Å, °)

*D*—H⋯*A*	*D*—H	H⋯*A*	*D*⋯*A*	*D*—H⋯*A*
N4—H4*A*⋯O1^i^	0.86 (3)	2.21 (2)	2.978 (4)	148 (3)
C17—H17*A*⋯O3	0.96	2.49	3.132 (7)	124

Symmetry code: (i) 
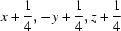
.

**Fig. 1.**
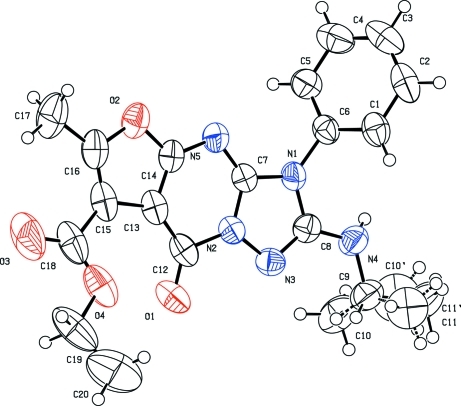
The molecular structure of the title compound, showing the atom-labeling scheme.

## supplementary crystallographic information

### Comment

Fused pyrimidine compounds are valued not only for their rich and varied chemistry, but also for many important biological properties. On the other hand, heterocycles containing triazoles nucleus also exhibit various biological activities.

The introduction of an triazole ring to the furopyrimidine system is expected to influence the biological activities significantly. As a part of our ongoing investigations on the preparation of derivatives of heterocyclic compounds (Ding *et al.*, (2004), Hu *et al.*, 2005, 2006, 2007, 2008), we have synthesized and structurally characterized characterized the title compound, and here we wish to report an X-ray crystal structure of it(Fig. 1).

In the molecule, the bond lengths and angles are unexceptional. In the title compound the ring system containing the three fused rings is essentially planar (r.m.s. deviation for all 12 non-H atoms 0.041 Å). The phenyl ring makes a dihedral angles of 54.41 (06)° with this ring system. The isopropyl group in molecule is disordered over two positions, with site occupancy factors 0.753 (9) and 0.247 (9); The structure is mainly stabilized by intermolecular weak N—H···O and intramolecular C—H···O hydrogen bonding interactions (Table 1) and π-π interactions with interplanar distances of 3.537Å between adjacent furan ring centroids (symmetry code: -*x*, -*y*, *z*) and 3.681Å between phenyl and pyrimidinone rings (symmetry code: 1/4 + *x*,1/4 - *y*,1/4 + *z*).

### Experimental

The title compound was obtained in excellent yield *via* aza-Wittig reaction. Crystals suitable for single-crystal X-ray diffraction were obtained by recrystallization from a mixed solvent of ethanol and dichloromethane (1:2 *v*/*v*) at room temperature.

### Refinement

In the absence of anomalous scatterers, 2307 Friedel pairs were merged. All H atoms were located in difference maps and treated as riding atoms, with C—H = 0.93 Å, *U*_iso_ = 1.2*U*_eq_ (C) for C*sp*^2^, C—H = 0.98 Å, *U*_iso_ = 1.2*U*_eq_ (C) for CH, C—H = 0.97 Å, *U*_iso_ = 1.2*U*_eq_ (C) for CH_2_, C—H = 0.96 Å, *U*_iso_ = 1.5*U*_eq_ (C) for CH_3_. The coordinates of the H atom bonded to N were refined with *U*_iso_ = 1.2*U*_eq_(N) and the N—H distance restrained to 0.86 (1) Å. The bond distances and 1–3 distances in the disordered groups were restrained to be equal within an effective e.s.d. of 0.01 Å.

### Crystal data

**Table:**

C_20_H_21_N_5_O_4_	*F*(000) = 3328
*M**_r_* = 395.42	*D*_x_ = 1.313 Mg m^−^^3^
Orthorhombic, *F**d**d*2	Mo *K*α radiation, λ = 0.71073 Å
Hall symbol: F 2 -2d	Cell parameters from 3861 reflections
*a* = 19.9810 (11) Å	θ = 2.2–20.8°
*b* = 37.3673 (19) Å	µ = 0.09 mm^−^^1^
*c* = 10.7181 (6) Å	*T* = 295 K
*V* = 8002.5 (7) Å^3^	Block, purple
*Z* = 16	0.20 × 0.20 × 0.10 mm

### Data collection

**Table:**

Bruker SMART 4K CCD area-detector diffractometer	2074 independent reflections
Radiation source: fine-focus sealed tube	1883 reflections with *I* > 2σ(*I*)
graphite	*R*_int_ = 0.042
φ and ω scans	θ_max_ = 27.0°, θ_min_ = 2.2°
Absorption correction: multi-scan (*SADABS*; Sheldrick, 2003)	*h* = −25→24
*T*_min_ = 0.981, *T*_max_ = 0.991	*k* = −44→47
21970 measured reflections	*l* = −13→13

### Refinement

**Table:**

Refinement on *F*^2^	Primary atom site location: structure-invariant direct methods
Least-squares matrix: full	Secondary atom site location: difference Fourier map
*R*[*F*^2^ > 2σ(*F*^2^)] = 0.043	Hydrogen site location: inferred from neighbouring sites
*wR*(*F*^2^) = 0.109	H atoms treated by a mixture of independent and constrained refinement
*S* = 1.09	*w* = 1/[σ^2^(*F*_o_^2^) + (0.0601*P*)^2^ + 2.4148*P*] where *P* = (*F*_o_^2^ + 2*F*_c_^2^)/3
2074 reflections	(Δ/σ)_max_ < 0.001
290 parameters	Δρ_max_ = 0.14 e Å^−^^3^
5 restraints	Δρ_min_ = −0.11 e Å^−^^3^

### Special details

**Table:**

Geometry. All e.s.d.'s (except the e.s.d. in the dihedral angle between two l.s. planes) are estimated using the full covariance matrix. The cell e.s.d.'s are taken into account individually in the estimation of e.s.d.'s in distances, angles and torsion angles; correlations between e.s.d.'s in cell parameters are only used when they are defined by crystal symmetry. An approximate (isotropic) treatment of cell e.s.d.'s is used for estimating e.s.d.'s involving l.s. planes.
Refinement. Refinement of *F*^2^ against ALL reflections. The weighted *R*-factor *wR* and goodness of fit *S* are based on *F*^2^, conventional *R*-factors *R* are based on *F*, with *F* set to zero for negative *F*^2^. The threshold expression of *F*^2^ > σ(*F*^2^) is used only for calculating *R*-factors(gt) *etc*. and is not relevant to the choice of reflections for refinement. *R*-factors based on *F*^2^ are statistically about twice as large as those based on *F*, and *R*- factors based on ALL data will be even larger.

### Fractional atomic coordinates and isotropic or equivalent isotropic displacement parameters (Å^2^)

**Table:**

	*x*	*y*	*z*	*U*_iso_*/*U*_eq_	Occ. (<1)
C1	0.25088 (16)	0.07862 (8)	0.2745 (4)	0.0618 (8)	
H1	0.2634	0.0694	0.1974	0.074*	
C2	0.29491 (17)	0.07774 (9)	0.3748 (4)	0.0743 (11)	
H2	0.3378	0.0685	0.3640	0.089*	
C3	0.2759 (2)	0.09026 (10)	0.4890 (4)	0.0829 (12)	
H3	0.3052	0.0889	0.5562	0.099*	
C4	0.2140 (2)	0.10476 (12)	0.5044 (4)	0.0831 (11)	
H4	0.2011	0.1133	0.5822	0.100*	
C5	0.17048 (18)	0.10680 (10)	0.4059 (3)	0.0678 (9)	
H5	0.1287	0.1173	0.4167	0.081*	
C6	0.18834 (15)	0.09340 (8)	0.2912 (3)	0.0520 (7)	
C7	0.07894 (14)	0.07677 (7)	0.1978 (3)	0.0487 (6)	
C8	0.14928 (15)	0.10258 (7)	0.0675 (3)	0.0517 (7)	
C9	0.21415 (19)	0.12881 (12)	−0.1012 (4)	0.0790 (11)	
H9A	0.1973	0.1093	−0.1537	0.095*	0.76
H9B	0.1720	0.1295	−0.1479	0.095*	0.24
C10	0.1741 (3)	0.16260 (18)	−0.1310 (7)	0.107 (2)	0.76
H10A	0.1276	0.1584	−0.1149	0.160*	0.76
H10B	0.1801	0.1687	−0.2173	0.160*	0.76
H10C	0.1896	0.1819	−0.0796	0.160*	0.76
C11	0.2851 (3)	0.1340 (3)	−0.1290 (7)	0.137 (3)	0.76
H11A	0.3032	0.1519	−0.0745	0.205*	0.76
H11B	0.2901	0.1416	−0.2141	0.205*	0.76
H11C	0.3086	0.1118	−0.1168	0.205*	0.76
C11'	0.2599 (11)	0.1007 (4)	−0.153 (2)	0.125 (8)	0.24
H11D	0.2996	0.0995	−0.1023	0.188*	0.24
H11E	0.2718	0.1067	−0.2367	0.188*	0.24
H11F	0.2376	0.0780	−0.1514	0.188*	0.24
C10'	0.2513 (12)	0.1649 (3)	−0.110 (3)	0.187 (17)	0.24
H10D	0.2212	0.1840	−0.0881	0.281*	0.24
H10E	0.2674	0.1684	−0.1932	0.281*	0.24
H10F	0.2885	0.1649	−0.0528	0.281*	0.24
C12	−0.00778 (15)	0.06210 (8)	0.0432 (3)	0.0575 (8)	
C13	−0.03791 (14)	0.04496 (8)	0.1483 (3)	0.0592 (8)	
C14	−0.00908 (14)	0.04888 (8)	0.2641 (3)	0.0549 (7)	
C15	−0.09993 (16)	0.02527 (8)	0.1723 (4)	0.0680 (10)	
C16	−0.10362 (17)	0.02052 (9)	0.2962 (4)	0.0715 (10)	
C17	−0.1520 (2)	0.00305 (13)	0.3833 (5)	0.0982 (14)	
H17A	−0.1864	−0.0087	0.3360	0.147*	
H17B	−0.1719	0.0209	0.4361	0.147*	
H17C	−0.1289	−0.0142	0.4336	0.147*	
C18	−0.1456 (2)	0.00781 (11)	0.0816 (5)	0.0832 (12)	
C19	−0.1552 (3)	−0.01350 (12)	−0.1254 (6)	0.1117 (18)	
H19A	−0.1946	0.0006	−0.1452	0.134*	
H19B	−0.1697	−0.0370	−0.0976	0.134*	
C20	−0.1128 (4)	−0.0169 (2)	−0.2349 (7)	0.158 (3)	
H20A	−0.0985	0.0064	−0.2614	0.237*	
H20B	−0.1375	−0.0282	−0.3009	0.237*	
H20C	−0.0743	−0.0312	−0.2147	0.237*	
N1	0.14054 (11)	0.09293 (6)	0.1915 (2)	0.0506 (6)	
N2	0.05524 (11)	0.07635 (6)	0.0793 (2)	0.0511 (6)	
N3	0.09879 (12)	0.09351 (6)	−0.0029 (2)	0.0538 (6)	
N4	0.20563 (14)	0.11862 (8)	0.0286 (3)	0.0636 (7)	
H4A	0.2249 (17)	0.1327 (8)	0.081 (3)	0.076*	
N5	0.04897 (13)	0.06436 (7)	0.2978 (2)	0.0568 (6)	
O1	−0.02832 (11)	0.06660 (6)	−0.0623 (2)	0.0728 (7)	
O2	−0.04849 (11)	0.03475 (6)	0.3546 (2)	0.0695 (6)	
O3	−0.20042 (15)	−0.00291 (9)	0.1061 (4)	0.1254 (13)	
O4	−0.11678 (14)	0.00387 (7)	−0.0285 (3)	0.0887 (9)	

### Atomic displacement parameters (Å^2^)

**Table:**

	*U*^11^	*U*^22^	*U*^33^	*U*^12^	*U*^13^	*U*^23^
C1	0.0516 (17)	0.0511 (16)	0.083 (2)	−0.0037 (13)	−0.0061 (16)	0.0058 (16)
C2	0.0521 (19)	0.0555 (18)	0.115 (3)	−0.0055 (15)	−0.021 (2)	0.016 (2)
C3	0.088 (3)	0.073 (2)	0.088 (3)	−0.015 (2)	−0.043 (2)	0.015 (2)
C4	0.094 (3)	0.091 (3)	0.065 (2)	−0.010 (2)	−0.020 (2)	0.0011 (19)
C5	0.063 (2)	0.079 (2)	0.062 (2)	−0.0057 (16)	−0.0053 (17)	0.0026 (16)
C6	0.0479 (16)	0.0494 (15)	0.0587 (17)	−0.0094 (13)	−0.0064 (13)	0.0071 (13)
C7	0.0428 (15)	0.0493 (15)	0.0540 (16)	−0.0008 (12)	−0.0032 (14)	−0.0011 (13)
C8	0.0534 (17)	0.0475 (15)	0.0542 (17)	0.0004 (13)	0.0045 (14)	−0.0061 (13)
C9	0.081 (2)	0.098 (3)	0.0576 (19)	−0.019 (2)	0.0117 (18)	−0.0011 (19)
C10	0.120 (5)	0.113 (5)	0.088 (4)	−0.002 (4)	0.018 (4)	0.035 (4)
C11	0.089 (4)	0.226 (10)	0.096 (5)	−0.018 (6)	0.039 (4)	0.026 (6)
C11'	0.18 (2)	0.093 (14)	0.105 (15)	−0.018 (15)	0.062 (16)	−0.013 (12)
C10'	0.26 (4)	0.110 (17)	0.19 (3)	0.06 (2)	0.16 (3)	0.081 (19)
C12	0.0508 (17)	0.0457 (16)	0.076 (2)	0.0031 (13)	−0.0168 (16)	−0.0029 (15)
C13	0.0449 (17)	0.0515 (17)	0.081 (2)	−0.0002 (13)	−0.0078 (16)	−0.0001 (16)
C14	0.0446 (15)	0.0510 (16)	0.0692 (19)	−0.0032 (13)	0.0058 (14)	−0.0018 (14)
C15	0.0450 (18)	0.0517 (18)	0.107 (3)	−0.0007 (14)	−0.0087 (18)	0.0031 (18)
C16	0.0485 (18)	0.0585 (19)	0.108 (3)	−0.0056 (14)	0.0049 (18)	−0.0043 (19)
C17	0.074 (3)	0.091 (3)	0.130 (4)	−0.023 (2)	0.029 (3)	−0.012 (3)
C18	0.058 (2)	0.061 (2)	0.131 (4)	−0.0096 (17)	−0.028 (2)	0.021 (2)
C19	0.120 (4)	0.063 (2)	0.152 (5)	−0.015 (2)	−0.081 (4)	0.011 (3)
C20	0.194 (7)	0.151 (5)	0.129 (5)	−0.003 (5)	−0.057 (5)	−0.036 (5)
N1	0.0410 (12)	0.0554 (13)	0.0552 (13)	−0.0055 (10)	−0.0011 (11)	0.0000 (11)
N2	0.0470 (13)	0.0505 (13)	0.0558 (14)	−0.0007 (10)	−0.0048 (11)	−0.0009 (11)
N3	0.0586 (15)	0.0519 (13)	0.0509 (13)	−0.0039 (11)	−0.0029 (12)	−0.0036 (11)
N4	0.0601 (16)	0.0739 (17)	0.0566 (16)	−0.0145 (14)	0.0082 (13)	−0.0032 (13)
N5	0.0484 (14)	0.0619 (15)	0.0601 (14)	−0.0057 (11)	0.0008 (12)	−0.0012 (12)
O1	0.0743 (15)	0.0658 (13)	0.0783 (16)	−0.0078 (11)	−0.0312 (13)	0.0074 (12)
O2	0.0551 (13)	0.0702 (14)	0.0832 (15)	−0.0106 (11)	0.0130 (12)	−0.0042 (12)
O3	0.0740 (18)	0.139 (3)	0.163 (3)	−0.0538 (19)	−0.031 (2)	0.036 (3)
O4	0.0692 (16)	0.0697 (16)	0.127 (3)	−0.0056 (13)	−0.0370 (18)	−0.0125 (16)

### Geometric parameters (Å, °)

**Table:**

C1—C6	1.378 (4)	C11'—H11D	0.9600
C1—C2	1.389 (5)	C11'—H11E	0.9600
C1—H1	0.9300	C11'—H11F	0.9600
C2—C3	1.364 (7)	C10'—H10D	0.9600
C2—H2	0.9300	C10'—H10E	0.9600
C3—C4	1.361 (6)	C10'—H10F	0.9600
C3—H3	0.9300	C12—O1	1.215 (4)
C4—C5	1.370 (5)	C12—N2	1.421 (4)
C4—H4	0.9300	C12—C13	1.429 (5)
C5—C6	1.374 (5)	C13—C14	1.376 (5)
C5—H5	0.9300	C13—C15	1.464 (5)
C6—N1	1.434 (4)	C14—N5	1.345 (4)
C7—N5	1.312 (4)	C14—O2	1.357 (4)
C7—N2	1.355 (4)	C15—C16	1.342 (6)
C7—N1	1.373 (3)	C15—C18	1.483 (6)
C8—N3	1.305 (4)	C16—O2	1.374 (4)
C8—N4	1.342 (4)	C16—C17	1.494 (6)
C8—N1	1.387 (4)	C17—H17A	0.9600
C9—N4	1.452 (5)	C17—H17B	0.9600
C9—C11	1.461 (7)	C17—H17C	0.9600
C9—C11'	1.496 (11)	C18—O3	1.196 (5)
C9—C10	1.528 (7)	C18—O4	1.321 (6)
C9—C10'	1.543 (11)	C19—O4	1.446 (5)
C9—H9A	0.9800	C19—C20	1.454 (9)
C9—H9B	0.9800	C19—H19A	0.9700
C10—H9B	1.2497	C19—H19B	0.9700
C10—H10A	0.9600	C20—H20A	0.9600
C10—H10B	0.9600	C20—H20B	0.9600
C10—H10C	0.9600	C20—H20C	0.9600
C11—H11A	0.9600	N2—N3	1.394 (3)
C11—H11B	0.9600	N4—H4A	0.86 (3)
C11—H11C	0.9600		
			
C6—C1—C2	118.9 (3)	C9—C10'—H10E	109.5
C6—C1—H1	120.6	H10D—C10'—H10E	109.5
C2—C1—H1	120.6	C9—C10'—H10F	109.5
C3—C2—C1	120.7 (3)	H10D—C10'—H10F	109.5
C3—C2—H2	119.7	H10E—C10'—H10F	109.5
C1—C2—H2	119.7	O1—C12—N2	120.1 (3)
C4—C3—C2	119.9 (4)	O1—C12—C13	130.8 (3)
C4—C3—H3	120.1	N2—C12—C13	109.1 (3)
C2—C3—H3	120.1	C14—C13—C12	119.1 (3)
C3—C4—C5	120.4 (4)	C14—C13—C15	104.5 (3)
C3—C4—H4	119.8	C12—C13—C15	136.1 (3)
C5—C4—H4	119.8	N5—C14—O2	118.4 (3)
C4—C5—C6	120.3 (4)	N5—C14—C13	130.4 (3)
C4—C5—H5	119.9	O2—C14—C13	111.1 (3)
C6—C5—H5	119.9	C16—C15—C13	106.7 (3)
C5—C6—C1	119.9 (3)	C16—C15—C18	123.8 (4)
C5—C6—N1	119.9 (3)	C13—C15—C18	128.8 (4)
C1—C6—N1	120.1 (3)	C15—C16—O2	110.9 (3)
N5—C7—N2	127.0 (3)	C15—C16—C17	135.3 (4)
N5—C7—N1	127.2 (3)	O2—C16—C17	113.8 (4)
N2—C7—N1	105.8 (2)	C16—C17—H17A	109.5
N3—C8—N4	125.8 (3)	C16—C17—H17B	109.5
N3—C8—N1	112.9 (2)	H17A—C17—H17B	109.5
N4—C8—N1	121.3 (3)	C16—C17—H17C	109.5
N4—C9—C11	110.1 (4)	H17A—C17—H17C	109.5
N4—C9—C11'	103.9 (10)	H17B—C17—H17C	109.5
N4—C9—C10	110.8 (4)	O3—C18—O4	123.9 (5)
C11—C9—C10	110.8 (6)	O3—C18—C15	124.5 (5)
C11'—C9—C10	145.3 (10)	O4—C18—C15	111.5 (3)
N4—C9—C10'	110.0 (11)	O4—C19—C20	108.0 (5)
C11'—C9—C10'	107.3 (9)	O4—C19—H19A	110.1
N4—C9—H9A	108.3	C20—C19—H19A	110.1
C11—C9—H9A	108.3	O4—C19—H19B	110.1
C10—C9—H9A	108.3	C20—C19—H19B	110.1
C10'—C9—H9A	141.5	H19A—C19—H19B	108.4
N4—C9—H9B	113.3	C19—C20—H20A	109.5
C11—C9—H9B	136.6	C19—C20—H20B	109.5
C11'—C9—H9B	110.9	H20A—C20—H20B	109.5
C10'—C9—H9B	111.1	C19—C20—H20C	109.5
C9—C10—H10A	109.5	H20A—C20—H20C	109.5
C9—C10—H10B	109.5	H20B—C20—H20C	109.5
H9B—C10—H10B	95.8	C7—N1—C8	105.9 (2)
C9—C10—H10C	109.5	C7—N1—C6	124.5 (2)
H9B—C10—H10C	147.0	C8—N1—C6	128.8 (2)
C9—C11—H11A	109.5	C7—N2—N3	111.6 (2)
C9—C11—H11B	109.5	C7—N2—C12	124.7 (3)
C9—C11—H11C	109.5	N3—N2—C12	123.6 (2)
C9—C11'—H11D	109.5	C8—N3—N2	103.7 (2)
C9—C11'—H11E	109.5	C8—N4—C9	120.9 (3)
H11D—C11'—H11E	109.5	C8—N4—H4A	116 (3)
C9—C11'—H11F	109.5	C9—N4—H4A	114 (3)
H11D—C11'—H11F	109.5	C7—N5—C14	109.0 (3)
H11E—C11'—H11F	109.5	C14—O2—C16	106.8 (3)
C9—C10'—H10D	109.5	C18—O4—C19	117.4 (4)
			
C6—C1—C2—C3	2.1 (5)	N4—C8—N1—C6	−8.7 (5)
C1—C2—C3—C4	−2.0 (5)	C5—C6—N1—C7	−56.1 (4)
C2—C3—C4—C5	0.2 (6)	C1—C6—N1—C7	120.2 (3)
C3—C4—C5—C6	1.6 (6)	C5—C6—N1—C8	135.5 (3)
C4—C5—C6—C1	−1.5 (5)	C1—C6—N1—C8	−48.2 (4)
C4—C5—C6—N1	174.8 (3)	N5—C7—N2—N3	176.9 (3)
C2—C1—C6—C5	−0.3 (5)	N1—C7—N2—N3	−3.0 (3)
C2—C1—C6—N1	−176.6 (3)	N5—C7—N2—C12	0.2 (5)
O1—C12—C13—C14	−168.6 (3)	N1—C7—N2—C12	−179.6 (2)
N2—C12—C13—C14	8.9 (4)	O1—C12—N2—C7	171.4 (3)
O1—C12—C13—C15	3.6 (6)	C13—C12—N2—C7	−6.5 (4)
N2—C12—C13—C15	−178.8 (3)	O1—C12—N2—N3	−4.9 (4)
C12—C13—C14—N5	−6.7 (5)	C13—C12—N2—N3	177.2 (2)
C15—C13—C14—N5	178.9 (3)	N4—C8—N3—N2	177.0 (3)
C12—C13—C14—O2	172.3 (3)	N1—C8—N3—N2	−1.2 (3)
C15—C13—C14—O2	−2.2 (3)	C7—N2—N3—C8	2.6 (3)
C14—C13—C15—C16	2.1 (4)	C12—N2—N3—C8	179.3 (2)
C12—C13—C15—C16	−170.9 (4)	N3—C8—N4—C9	2.3 (5)
C14—C13—C15—C18	−168.0 (3)	N1—C8—N4—C9	−179.6 (3)
C12—C13—C15—C18	19.0 (6)	C11—C9—N4—C8	−160.5 (6)
C13—C15—C16—O2	−1.3 (4)	C11'—C9—N4—C8	−103.2 (11)
C18—C15—C16—O2	169.4 (3)	C10—C9—N4—C8	76.5 (5)
C13—C15—C16—C17	−179.8 (4)	C10'—C9—N4—C8	142.2 (10)
C18—C15—C16—C17	−9.1 (7)	N2—C7—N5—C14	3.4 (4)
C16—C15—C18—O3	23.7 (6)	N1—C7—N5—C14	−176.8 (3)
C13—C15—C18—O3	−167.7 (4)	O2—C14—N5—C7	−179.0 (3)
C16—C15—C18—O4	−153.1 (4)	C13—C14—N5—C7	−0.1 (5)
C13—C15—C18—O4	15.5 (5)	N5—C14—O2—C16	−179.5 (3)
N5—C7—N1—C8	−177.7 (3)	C13—C14—O2—C16	1.4 (3)
N2—C7—N1—C8	2.1 (3)	C15—C16—O2—C14	0.0 (4)
N5—C7—N1—C6	11.6 (5)	C17—C16—O2—C14	178.9 (3)
N2—C7—N1—C6	−168.6 (2)	O3—C18—O4—C19	2.0 (6)
N3—C8—N1—C7	−0.5 (3)	C15—C18—O4—C19	178.8 (3)
N4—C8—N1—C7	−178.8 (3)	C20—C19—O4—C18	−176.6 (4)
N3—C8—N1—C6	169.5 (3)		

### Hydrogen-bond geometry (Å, °)

**Table:**

*D*—H···*A*	*D*—H	H···*A*	*D*···*A*	*D*—H···*A*
N4—H4A···O1^i^	0.86 (3)	2.21 (2)	2.978 (4)	148 (3)
C17—H17A···O3	0.96	2.49	3.132 (7)	124.

Symmetry codes: (i) *x*+1/4, −*y*+1/4, *z*+1/4.
